# Hyperkeratotic lesions on palms and soles

**DOI:** 10.1016/j.jdcr.2024.06.028

**Published:** 2024-07-06

**Authors:** Usha N. Khemani, Neha Fogla, Sushma Poojary, Avinash A. Sajgane

**Affiliations:** Department of Dermatology, Venereology and Leprosy, Grant Government Medical College and Sir JJ Group of Hospitals, Mumbai, India

**Keywords:** arsenicosis, ayurvedic medication, keratosis, melanosis, premalignant

## Case presentation

**Figure 1:**
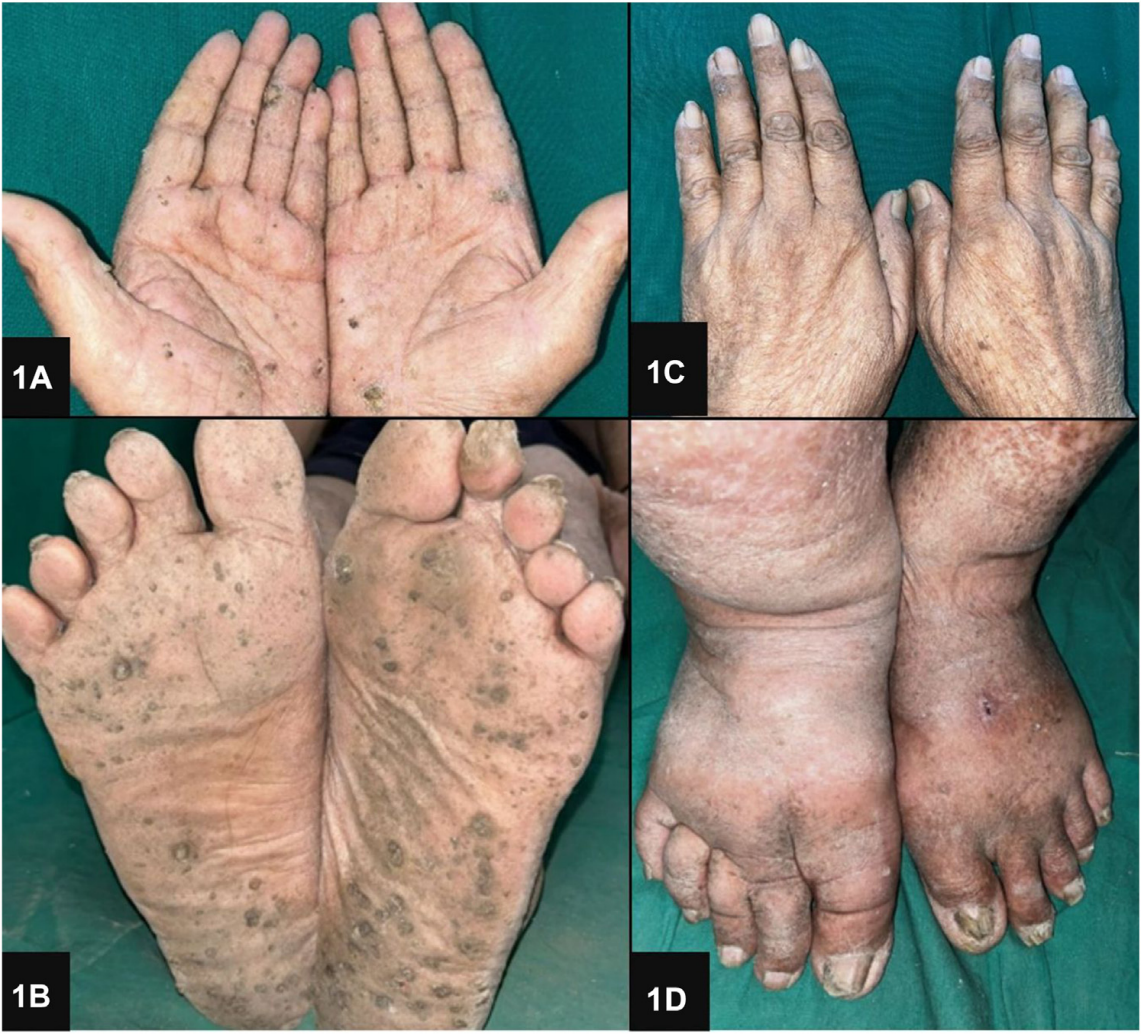

**Figure 2:**
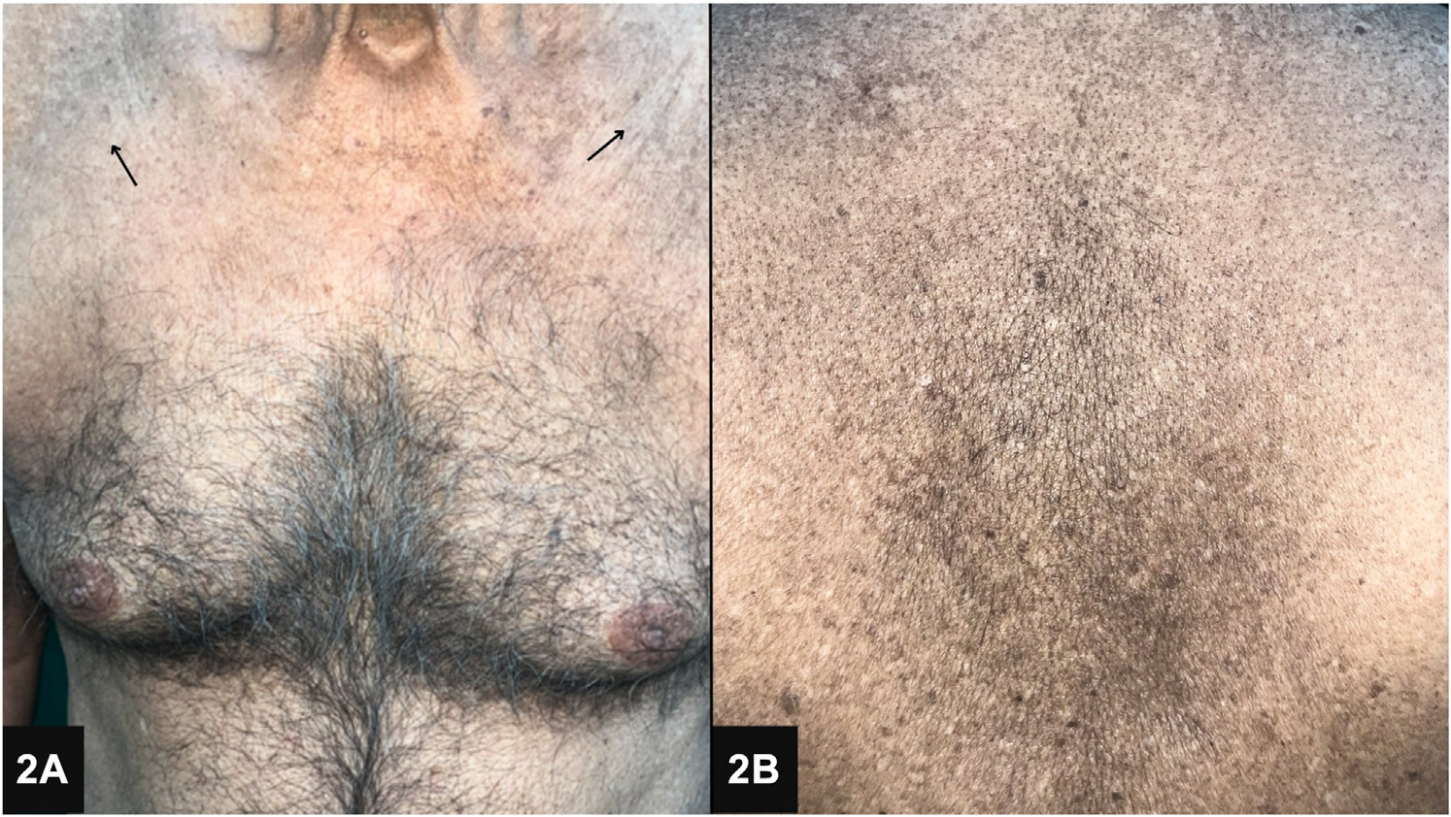

**Figure 3:**
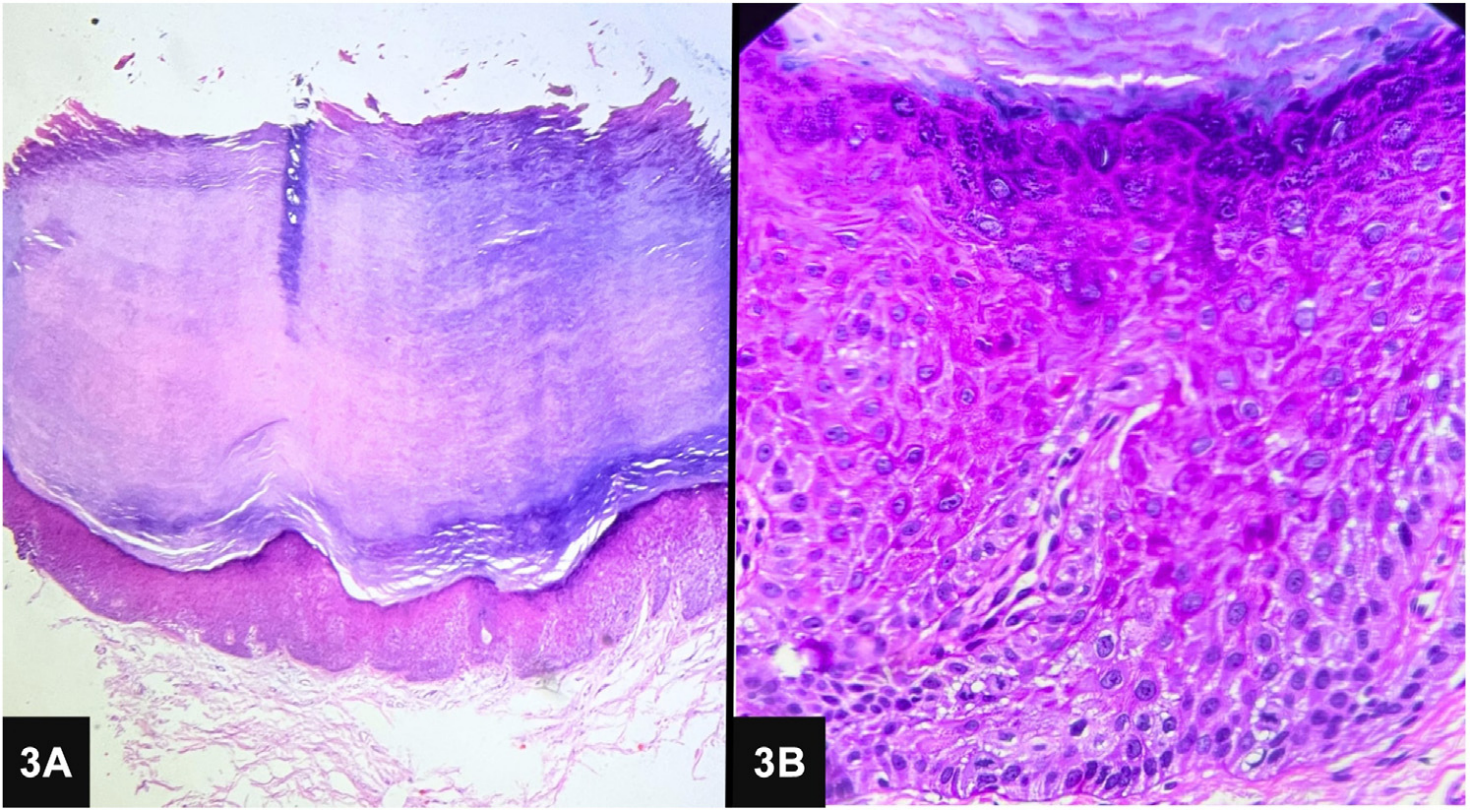

A 74-year-old man presented with an 8-year history of hyperkeratotic, nontender verrucous papules and plaques on the palms and soles (Fig 1). The patient reported mild pain and itching on the manipulation of lesions. He had been self-administering “Smriti Sagar Ras,” an ayurvedic medication, for the past 15 years as an immunity booster. Previous surgical removal of the lesions resulted in recurrence. On examination, diffuse areas of hyperpigmentation were noted on the abdomen, chest, and back (Fig 2). Skin biopsy showed compact marked hyperkeratosis, parakeratosis, hypergranulosis, acanthosis, and dyskeratotic cells in the epidermis (Fig 3).

**Question 1: What is the possible diagnosis in this case?**

A. Poikilodermatous mycosis fungoides
B Dyskeratosis congenita
C. Arsenicosis
D. Epidermodysplasia verruciformis
E. Dyschromatosis symmetrica hereditaria

**Answers:**

A. Poikilodermatous mycosis fungoides – Incorrect. Poikilodermatous mycosis fungoides, a variant of cutaneous T-cell lymphoma, typically presents with poikiloderma (a combination of hypopigmentation, hyperpigmentation, telangiectasia, and atrophy). The hyperkeratotic lesions and specific history of ayurvedic medication use in this case do not align with this diagnosis.
B. Dyskeratosis congenita – Incorrect. Dyskeratosis congenita is a rare genetic disorder characterized by a triad of abnormal-appearing skin pigmentation, nail dystrophy, and leukoplakia. Although dyskeratosis is noted in the biopsy, the patient’s age of onset and specific lesion morphology are not consistent with this condition.
C. Arsenicosis – Correct. The patient’s chronic ingestion of “Smriti Sagar Ras,” which contains purified arsenic, and the clinical presentation of hyperkeratotic, verrucous papules and plaques on the palms and soles indicate arsenicosis.^1^ Additional findings, such as dyschromia, align with this diagnosis. Arsenicosis typically presents with palmoplantar hyperkeratosis, melanosis, and can lead to various malignancies and systemic symptoms.
D. Epidermodysplasia verruciformis – Incorrect. Epidermodysplasia verruciformis is a rare genetic disorder predisposing individuals to widespread human papillomavirus infections, leading to wart-like lesions and a high risk of malignancy. The patient’s age of onset and specific lesion distribution do not fit this diagnosis.
E. Dyschromatosis symmetrica hereditaria – Incorrect. Dyschromatosis symmetrica hereditaria is characterized by a mixture of hyperpigmented and hypopigmented macules that primarily affect the dorsal aspects of the hands and feet, typically appearing in early childhood. The skin changes in dyschromatosis symmetrica hereditaria are usually symmetric and confined to the extremities, without the diffuse hyperpigmentation and hyperkeratotic lesions seen in this patient’s case.

**Question 2: What is the next best step in diagnosis?**

A. Patch testing
B. Blood arsenic levels
C. Blood, hair, and nail tests
D. Complete blood count with peripheral smear
E. Liver function tests

**Answers:**

A. Patch testing – Incorrect. Allergy patch testing diagnoses allergic contact dermatitis, not systemic conditions such as arsenicosis.
B. Blood arsenic levels – Incorrect. Relying solely on blood arsenic levels is inadequate for diagnosing arsenicosis, as there could be false negatives resulting from prolonged consumption of low amounts of arsenic.
C. Blood, hair, and nail tests – Correct. Measurement of arsenic levels in blood, hair, and nails can confirm chronic arsenic exposure, supporting the diagnosis of arsenicosis.^2^ Our patient underwent the same and had raised arsenic in all the samples. Hair analysis revealed an arsenic content of 0.29 μg/g (normal, <0.08 μg/g), whereas nail arsenic levels were elevated at 4.56 μg/g (normal, 0.2-3.0 μg/g). Blood arsenic levels were also increased at 8.23 μg/dL (normal, 0.17-5 μg/dL).
D. Complete blood count with peripheral smear – Incorrect. A complete blood count may show nonspecific abnormalities but would not confirm arsenicosis.
E. Liver function tests – Incorrect. Although liver function tests may show abnormalities in arsenicosis, they do not directly measure arsenic levels in the body.

**Question 3: Which form of arsenic predominantly causes toxic effects?**

A. Pentavalent arsenate (As5+)
B. Trivalent meta-arsenite (As3+)
C. Organic arsenic
D. Arsenic disulfide
E. Arsenic-containing minerals

**Answers:**

A. Pentavalent arsenate (As5+) – Incorrect. Although arsenic is primarily consumed in its pentavalent form (arsenate), the trivalent form is more predominant in toxic effects after reduction via glutathione.^3^
B. Trivalent meta-arsenite (As3+) – Correct. The toxic effects of arsenic are primarily mediated by the trivalent form, which binds to sulfhydryl groups in essential compounds, leading to various toxicities including skin manifestations. Arsenic induces apoptosis through free radical generation, and its cutaneous toxicity is linked to its effect on various cytokines, growth factors, and transcription factors. Increased expression of cytokeratins such as k16 and k8/k18 correlate with the histopathologic findings of hyperkeratosis and dysplasia respectively.^4^
C. Organic arsenic – Incorrect. Organic arsenic compounds are generally less toxic than inorganic forms and are less relevant to this case.
D. Arsenic disulfide – Incorrect. Arsenic disulfide is not the primary form of arsenic implicated in this case.
E. Arsenic-containing minerals –Incorrect. Arsenic is naturally present in various minerals, such as arsenopyrite and realgar, which can release arsenic into the environment through weathering and mining. This is not the main form of arsenic that causes toxicity.

## Conflicts of interest

None disclosed.
